# First Human Cases of Tickborne Encephalitis, Norway

**DOI:** 10.3201/eid1012.040598

**Published:** 2004-12

**Authors:** Tone Skarpaas, Unn Ljøstad, Anders Sundøy

**Affiliations:** * Sørlandet Hospital Kristiansand, Kristiansand, Norway

**Keywords:** tickborne encephalitis, Norway, human cases, seroprevalence, dogs, dispatch

## Abstract

The first reported case of tickborne encephalitis (TBE) in Norway occurred in 1997. From 1997 to 2003, from zero to two cases of human TBE have been diagnosed per year in Norway, for a total of eight cases. Clinical TBE cases in dogs are not reported in Norway.

In Scandinavia, tickborne encephalitis (TBE) is endemic in the coastal areas along the Baltic Sea. The first reports of TBE from Sweden and Finland date back to1954 and 1956, but the disease was not been found in Norway until 1997. Since then, eight cases of human TBE have been reported, and five cases have been published in a Norwegian journal (*1**,**2*).

In a study of serum samples from dogs in Aust-Agder County, immunoglobulin (Ig) G antibodies to TBE virus (TBEV) were detected in 16.4% of the samples (*3*). Clinical TBE cases in dogs are not reported in Norway, but the disease is probably underdiagnosed because antibody testing is not usually done. We present three new cases of human TBE and summarize the clinical characteristics and laboratory findings from all eight patients.

## The Study

Patient 6 is a 62-year-old man from the town of Mandal who was bitten by a tick; onset of symptoms began 2 weeks later. At the end of May, the patient was dizzy and weak, had a headache, chills, and fever. He was hospitalized on June 11, 2002.

The antibody from serum sample profiles showed previous infection with herpes simplex and varicella zoster viruses. *Borrelia* antibodies could not be detected in serum samples taken at 5-week intervals. IgM antibodies against *Mycoplasma pneumoniae* were not detected, and virus cultures were negative. Nucleic acids from herpes simplex virus, varicella-zoster virus, or enterovirus were not detected in cerebrospinal fluid (CSF).

TBEV IgM and IgG antibodies were detected in serum samples, with high levels of IgM (optical density [OD] 1.580 on June 13 to OD 0.899 on July 18) and high IgG levels (13.06, OD 1.235 on June 13 to OD 1.742 on July 18). Cut-off values were 0.250 for IgM and 0.263 for IgG on June 13 and 0.271 for IgM and 0.278 for IgG on June 18. Neutralization test antibodies in serum samples rose from <5 in samples taken on June 13 to 10 in samples from July 18. Symptoms gradually disappeared, and the patient completely recovered in 2 months.

Patient 7 is a 53-year-old man who was visiting a cabin in the coastal area near Mandal. Symptoms began at the end of June, with fever, increasing headache, nausea, and vomiting. He was hospitalized on July 20, 2002. His liver enzymes were slightly raised. Computed tomographic scan was normal. *Borrelia burgdorferi* antibodies were detected in serum, without intrathecal production of *Borrelia* antibodies. Nucleic acids from herpes simplex virus, varicella-zoster virus, or enterovirus were not detected in CSF.

TBEV IgM and IgG antibodies were detected in serum samples, with high levels of IgM (OD 2.064 on July 22; OD 1.916 on July 30; and OD 1.499 on August 8) and rising IgG levels (OD 0.597 on July 22; OD 0.876 on July 30; and OD 1.993 on August 8). Cut-off values were 0.277–0.280 for IgM and 0.266–0.275 for IgG). Neutralization test antibody levels rose from <5 in serum taken July 21 to 10 in serum from November 25. Borderline values of TBEV antibodies were found in spinal fluid. During the first several months after illness onset, the patient had cognitive dysfunction but gradually returned to work.

Patient 8 is a 74-year-old man, who lives in Kristiansand and has a camper in Søgne. Since August 2003, he had increasing headache and from October 3, 2003 the headache was intense and accompanied by nausea and vomiting. His personality was altered during these weeks, with reduced memory about recent events in particular, irritability, and verbal aggressiveness. He was admitted to the hospital on October 6, 2003. Results from computed tomography were normal, and electroencephalogram showed changes consistent with encephalitis. *Borrelia* antibody levels in serum samples were low. Intrathecal production of *Borrelia* antibodies could not be detected. Nucleic acids from herpes simplex or enterovirus were not detected in spinal fluid.

High levels of TBEV IgM (OD 1.461 on October 6 and OD 1.200 on November 5) were detected in sera together with rising IgG levels (OD 0.652 on October 6 and OD 1.475 on November 11). Cut-off values were 0.281–0.286 for IgM and 0.259–0.265 for IgG. In spinal fluid from October 3, intrathecal production of TBEV antibodies could not be detected, but one month later, intrathecal IgM was produced. During hospitalization, the patient recovered well. After 10 to 11 days, he was aware, and his mental situation improved considerably. He was also able to walk on stairs. After 4 to 5 months, he was fully recovered.

The Agder counties have the highest incidence of *Borrelia* infections in Norway (33 cases/100,000 persons, 1997–2003). The incidence of neuroborreliosis is 10 cases per 100,000 persons (*4*). The first case of TBE in Norway was reported in 1997 (*1*). The previously published clinical signs and symptoms and results from these five patients (*1**,**2*) are summarized as case 1–5, while the three new patients are presented as patients 6–8 (Table 1 and Table 2).

**Table 1:** Characteristics of patients with tickborne encephalitis, Norway, 1997–2003

Date	Patient no.	Age	Sex	Symptoms/neurologic disturbances	Disease duration
08/1997	1	42	Male	Biphasic course. Headache, nausea, vomiting, migrating myalgia/hyperreflexia.	1 mo
08/1998	2	72	Male	Fever, nausea, vomiting, confusion, speech disturbance/somnolence, mental disturbance, vertigo, bilateral ptosis, paresis of eye muscles, light throat paresis, paresis of the left shoulder	Sequela >1 y
10/1999	3	60	Male	Fever, headache/normal organ status	1 month
10/2000	4	67	Male	Fever, headache, nausea, vomiting/confusion, cognitive dysfunction.	Cognitive dysfunctions in 2–3 mo
10/2000	5	43	Female	Biphasic course. Fever, headache, nausea, vomiting/diplopia, ataxia.	1 mo
06/2002	6	62	Male	Fever, headache, nausea/ataxia.	2 mo
07/2002	7	53	Male	Fever, headache, nausea, vomiting/paresthesia, ataxia	Cognitive dysfunctions in months
10/2003	8	74	Male	Headache, nausea, vomiting, altered personality, irritability, verbal aggressiveness/confusion, and ataxia.	4–5 mo

**Table 2:** Laboratory findings in serum and spinal fluid specimens from patients with tickborne encephalitis, Norway, 1997–2003^a^

Patient no.	Temperature °C	Serum		Spinal fluid	
		CRP mg/l	Pleocytosis 10^9^/L	Pleocytosis 10^6^/L	Protein mg/L
1	38.4	10	ND	ND	ND
2	39.6	105	8,3	500	850
3	–	–	_–	47	790
4	39.6	32	11,3	39	622
5	40	15	8,6	24	609
6	39.5	18	13	130	1180
7	38	46	15,4	115	1337
8	38.3	15	12,1	22	649

^a^ND, not done. TBE was diagnosed retrospectively. The patient was not hospitalized; –, not published.

The eight patients included seven men and one woman from 42 years to 74 years of age. Biphasic courses were described in two patients. All patients had intense headache, seven had vertigo and nausea, and six had vomiting. Seven patients were hospitalized, three with reduced consciousness, two with mental disturbances; all seven had more or less severe neurologic abnormalities. Three had ataxia; one had diplopia; and one had speech difficulties, bilateral ptosis, paresis of eye and pharynx muscles, and paresis of muscles in the left shoulder. One had an epileptic seizure. All patients had fever, with temperatures from 38°C to 40°C. Serum samples were obtained from all eight patients and had signs of inflammation with C-reactive protein level of 10–105 mg/L and elevated leukocyte count of 8.3–15.4 x 10^9^/L. Seven patients underwent lumbar puncture; CSF pleocytosis and elevated protein levels were found in all patients. Nucleic acids from herpes simplex virus, varicella-zoster virus, or enterovirus were not detected in the spinal fluid specimens, which excludes the most common differential diagnostic causes of encephalitis.

In all patients, high serum levels of TBEV IgM and IgG antibodies were detected with enzyme-linked immunosorbent assay methods. In neutralization tests, serum antibody titers increased from <5 to 10 in five of the patients, 10 to 20 in one, and 10 to 40 in one patient (*1**,**2*). Seven patients recovered during the first 6 months. Two had cognitive dysfunctions during the first several months. One person still had paresis and atrophy of the shoulder muscles 1 year later.

Although the diagnostic tests are not absolutely specific for TBE compared to the closely related Louping ill virus, no cases of Louping ill virus in livestock (Snorre Stuen, pers. comm.) or human infections have been reported in Norway since 1991; none of the eight patients lived close to or worked with sheep or goats. The clinical characteristics of the Norwegian patients are similar to those of Swedish patients (*5*). In Sweden, the disease is caused by TBEV subtype 1.

All eight patients with TBE in Norway became ill after being bitten by a tick in the coastal areas of the Agder counties. Four had been on Tromøy Island in Aust-Agder County before becoming ill, while one had been in Lyngdal and three in Mandal and Søgne in Vest-Agder County. None of the patients had been abroad in the 3 weeks before becoming ill.

TBE was assumed not to be present in Norway. Thus, all patients with suspicious cases of TBE may not have been tested for antibodies to TBEV. In Agder, we have tested for TBE since 1999, but the disease may still be underdiagnosed.

Some seroprevalence studies have been carried out. TBEV IgG antibodies were detected in 0.3% to 0.4% of the serum samples from persons in Agder counties. From persons on Tromøy Island, antibodies were found in 2.4% of serum samples, and in other coastal districts, the seroprevalence was 0%–11% (*1**,**6*). The number of human serum samples tested is limited, and the vaccination status is unknown. However, vaccination is unlikely because Norwegians are only vaccinated against flaviviruses on special travel indications.

In Sweden, the incidence of human cases of TBE has risen during the last few years, and new TBE foci have been reported (*7*). During the last 2 decades, an increased number of TBE cases have been reporting in most European countries. Changes in the distribution of TBEV have been indicated, and the Norwegian cases are from areas where new foci have been predicted (*8*).

## Conclusions

In Norway, 0–2 cases of TBE were diagnosed per year from 1997 to 2003. All patients have been bitten by a tick in the Agder counties in southern Norway. Of the first eight Norwegian patients, four had been on Tromøy Island in Aust-Agder County before becoming ill. The four most recent patients were bitten by ticks in Lyngdal, Mandal, and Søgne in the coastal areas of Vest-Agder County. The seroprevalence studies indicate that Tromøy and some spots along the coast in the southernmost part of Vest-Agder County may have a higher incidence of TBE than the rest of the Agder counties.

Our results confirm that TBE occurs in the coastal areas of southern Norway. Although TBE is a rare disease in Norway, the situation has to be monitored carefully. Further studies are required to establish guidelines for preventive measures such as vaccination.
